# Deletion of *wfs1* Impairs Oligodendrocyte Precursor Cells Dorsal Distribution and Myelination Through the *wfs1*-*hmgcs1* Axis in Zebrafish

**DOI:** 10.3390/biology15161402

**Published:** 2026-08-16

**Authors:** Xiahui Tang, Ziang Zhao, Kunlun Yao, Dinggang Fan, Keqiang Li, Junhui Zhou, Zongyi Wang, Bing Hu

**Affiliations:** 1 Center for Advanced Interdisciplinary Science and Biomedicine of Institute of Health and Medicine, Division of Life Sciences and Medicine, University of Science and Technology of China, Hefei 230026, China; tangxiahui@mail.ustc.edu.cn (X.T.); zhaoza@mail.ustc.edu.cn (Z.Z.); ykl2003@mail.ustc.edu.cn (K.Y.); fdg@mail.ustc.edu.cn (D.F.); likeqiang@mail.ustc.edu.cn (K.L.); zjh23008001@mail.ustc.edu.cn (J.Z.); 2 Department of Psychiatry and Behavioral Sciences, Stanford University School of Medicine, Stanford, CA 94305, USA

**Keywords:** Wolfram syndrome, *wfs1*, oligodendrocyte precursor cells, dorsal distribution, myelination, *hmgcs1*, zebrafish, mevalonate pathway, isoprenylation

## Abstract

Wolfram syndrome (WS) is a neurodegenerative disorder caused by mutations in the endoplasmic reticulum (ER) transmembrane protein WFS1. Mutations in *WFS1* lead to ER stress and dysregulated calcium signaling, resulting in progressive neurological dysfunction. Myelination is a core pathological process in various central nervous system (CNS) diseases. However, the role of *WFS1* in oligodendrocyte development and myelination remains unknown. Here, using CRISPR-Cas9-generated *wfs1a/wfs1b* double-knockout (*wfs1^−/−^*) zebrafish, we demonstrated that *wfs1* deficiency significantly delayed the dorsal distribution of oligodendrocyte precursor cells (OPCs) along Mauthner axons. Furthermore, *wfs1* mutation was associated with early hypomyelination of axons at 5 dpf, with partial recovery observed by 8 dpf. Mechanistically, combined transcriptomic and pharmacological analysis revealed that *wfs1* mutation led to the suppression of the steroid biosynthesis pathway through downregulation of *hmgcs1*, which encodes the upstream condensation enzyme of the mevalonate pathway and supplies substrate for the downstream rate-limiting enzyme HMGCR, thereby potentially reducing isoprenoid precursor availability. Pharmacological supplementation with geranylgeraniol (GGOH) specifically rescued dorsal distribution defects, supporting involvement of the isoprenylation pathway, although direct regulation of *hmgcs1* by *wfs1* and complete rescue of myelin structure require further mechanistic validation. Taken together, our study supports a novel *wfs1*-*hmgcs1* axis, which may regulate dorsal distribution and myelination through the isoprenylation pathway; notably, classical ER stress markers were also concurrently upregulated in *wfs1^−/−^* larvae, although whether the isoprenylation deficiency and ER stress act independently or synergistically remains to be determined. These findings provide new insights into WS-associated early hypomyelination and suggest *hmgcs1* as a potential therapeutic target for myelin defects caused by WS.

## Simple Summary

Wolfram syndrome, a rare autosomal recessive neurodegenerative disorder characterized by progressive neuronal degeneration, was investigated in this study. We demonstrate that suppression of *hmgcs1* expression is associated with *wfs1* deficiency and may contribute to impaired myelination, suggesting the *wfs1*-*hmgcs1* axis as a potential therapeutic target for WS-related hypomyelinating conditions that warrants further mechanistic validation.

## 1. Introduction

Wolfram syndrome (WS) is a rare autosomal recessive neurodegenerative disorder characterized by progressive neuronal degeneration. Its core neuropathological manifestations include diabetes insipidus, diabetes mellitus, optic atrophy, and deafness [1,2,3,4,5]. The disease is primarily caused by mutations in the *WFS1* gene, which encodes an endoplasmic reticulum (ER)-resident protein called wolframin, known to regulate calcium homeostasis and apoptosis [6,7,8]. *WFS1* mutations are heterogeneous, encompassing missense, nonsense, frameshift, and splice-site variants, with the majority located in the transmembrane domain encoded by exon 8. Most disease-causing mutations result in complete or near-complete loss of wolframin function, though some missense variants may retain partial protein activity [4,9]. Mutations in *WFS1* lead to ER stress and dysregulated calcium signaling, resulting in multisystem neurological disorders [9,10]. Beyond the classical symptoms, WS is often accompanied by neurological manifestations including central respiratory failure, psychiatric disorders, and motor dysfunction, resulting in most patients having a lifespan of no more than 30 years [10,11].

Neuroimaging studies have provided important evidence for white matter abnormalities in WS patients. Diffusion tensor imaging (DTI) studies have revealed reduced fractional anisotropy and increased radial diffusivity in major white matter tracts, consistent with impaired axonal myelination [2]. Longitudinal analyses indicate that these white matter changes are present from early childhood and are more consistent with developmental hypomyelination than with secondary hypomyelination [12]. Furthermore, gene expression analyses in WS patient-derived tissues have identified dysregulation of oligodendrocyte-related genes, suggesting a direct role of WFS1 in oligodendrocyte biology [12].

Myelination plays an important role in various central nervous system (CNS) diseases [13,14]. Spatiotemporal differentiation and migration of oligodendrocyte precursor cells (OPCs) are prerequisites for myelination in CNS [15,16]. OPCs are widely distributed throughout the CNS and exhibit high migratory and proliferative capacities [17,18]. Their differentiation is regulated by metabolic reprogramming pathways, such as cholesterol and steroid synthesis, which directly drive OPCs maturation by modulating cytoskeletal dynamics and membrane lipid assembly [19,20]. Impaired myelin integrity is closely associated with OPC dysfunction in neurodegenerative diseases [21,22,23]. Notably, *WFS1* is highly expressed in oligodendrocytes, and its genetic polymorphisms significantly correlate with myelin integrity in neurodegenerative disorders [24,25]. Animal studies further suggest that *wfs1* deficiency causes neuronal defects in zebrafish [26,27] and myelin disorganization in the mouse brainstem [28,29]. However, the roles and downstream mechanisms of *wfs1* in regulating dorsal distribution and myelination are largely unknown.

The mevalonate pathway is a central metabolic hub that converts acetyl-CoA into diverse lipid products essential for cell function. HMGCS1 (3-hydroxy-3-methylglutaryl-CoA synthase 1) catalyzes the condensation of acetyl-CoA to generate HMG-CoA, providing substrate for the rate-limiting enzyme HMGCR. Downstream products of the mevalonate pathway include cholesterol, a key structural component of myelin membranes, as well as farnesyl pyrophosphate (FPP) and geranylgeranyl pyrophosphate (GGPP), which serve as lipid donors for protein prenylation. Protein isoprenylation is a post-translational modification that enables membrane anchoring and functional activation of small GTPases such as Rho, Rac, and Ras family members, which are critical regulators of cytoskeletal dynamics and cell migration. Geranylgeraniol (GGOH) is a natural isoprenoid alcohol that can be phosphorylated intracellularly to generate GGPP, thereby bypassing upstream mevalonate pathway steps and directly supplying substrate for geranylgeranylation. Previous studies in zebrafish have demonstrated that hmgcs1 mutation impairs dorsal distribution and myelin gene expression through disruption of protein isoprenylation, and these defects can be rescued by GGOH supplementation [30].

Zebrafish have become an ideal model for studying dorsal distribution and early myelin development because their larvae are transparent, which provides conditions for real-time dynamic tracking of fluorescent proteins at single cell level in vivo [31,32,33,34]. Mauthner cells (M-cells) are a pair of central neurons located in the hindbrain of zebrafish; their axons exhibit evolutionarily conserved myelination and are among the first to be myelinated during development [31,35,36,37], offering a spatiotemporally precise model for studying dorsal distribution and oligodendrocyte lineage development [31,37,38]. Given the potential role of *wfs1* in ER lipid metabolism [5], we hypothesized that *wfs1* deficiency may modulate OPC development via *hmgcs1*-dependent metabolic reprogramming.

In this study, using in vivo imaging of *wfs1a*/*wfs1b* double-knockout (*wfs1^−/−^*) zebrafish larvae constructed by CRISPR-Cas9 technology to investigate the role of *wfs1* in dorsal distribution, we found that *wfs1* deficiency significantly delayed the dorsal distribution of Mauthner cell-associated OPCs and downregulated the expression of myelin genes (*mbp*, *mag*, *mpz*, *plp1b*). Using transmission electron microscopy, we further demonstrated that *wfs1* mutation leads to early hypomyelination of axons at 5 dpf, with partial recovery by 8 dpf. Mechanistically, RNA sequencing (RNA-seq) analysis further revealed suppression of the steroid synthesis pathway, especially the downregulation of *hmgcs1*, a key enzyme in the mevalonate pathway, which may directly regulate cell migration efficiency and differentiation. Pharmacological treatment with geranylgeraniol (GGOH), an isoprenoid precursor, specifically rescued the dorsal distribution defects, indicating that *wfs1* deficiency is associated with dorsal distribution impairment through *hmgcs1*-dependent metabolic reprogramming.

Together, our study supports the metabolic regulatory association of the *wfs1*–*hmgcs1* axis in myelination, providing a novel potential therapeutic target for WS-related myelin defects. These findings also provide new insight into promoting myelin regeneration and oligodendrocyte protection.

## 2. Materials and Methods

### 2.1. Zebrafish Strains and Rearing

Wild-type (WT/AB) zebrafish and the transgenic strain Tg (*olig2*: EGFP) zebrafish, which drives EGFP expression in OPCs [39], were used in this study. Zebrafish were maintained in a recirculating aquatic system at a constant temperature of 28.5 ± 0.5 °C, with a 14 h/10 h light/dark cycle. Embryos were incubated in E3 medium (5 mM NaCl, 0.17 mM KCl, 0.33 mM CaCl_2_, 0.33 mM MgSO_4_; all chemicals from Sangon Biotech, Shanghai, China) supplemented with 0.1% methylene blue (Sigma-Aldrich, St. Louis, MO, USA) to inhibit fungal growth [40]. Starting at 24 h post-fertilization (hpf), embryos/larvae were continuously exposed to E3 medium containing 0.003% N-phenylthiourea (PTU, Sigma-Aldrich, St. Louis, MO, USA) to suppress melanogenesis. All animal procedures were approved by the Institutional Animal Care and Use Committee of the University of Science and Technology of China (protocol code: USTCACUC29120423098, Hefei, China). Animals were maintained and experiments were conducted in compliance with institutional guidelines for the care and use of laboratory animals.

### 2.2. Generation of wfs1^−/−^ Zebrafish Strain

The heritable *wfs1a* mutant zebrafish line was generated using CRISPR-Cas9-mediated genome editing. A target-specific single-guide RNA (sgRNA) targeting the sequence 5′-GGATAATAGTGCTCAGCCAC-3′ (PAM: TGG) was designed to the sixth exon of the *wfs1a* gene using the CRISPOR online design tool to ensure high efficiency and specificity [41]. The sgRNA was synthesized via in vitro transcription (MEGAshortscript T7 kit, Thermo Fisher Scientific, Waltham, MA, USA) and purified. The purified sgRNA (300 ng/µL) was complexed with Cas9 protein (300 ng/µL; PC1350, Inovogen, Chongqing, China) and 1 nL of the mixture was microinjected into the yolk of one-cell-stage embryos of WT/AB strain zebrafish to generate F0 mutations. Injected embryos were raised to adulthood and screened by fin-clip genotyping. F0 founders carrying germline mutations were outcrossed to WT/AB fish to generate F1 heterozygotes, which were subsequently intercrossed to produce F2 homozygotes. Genotyping was performed by PCR amplification of the target region using primers *wfs1a-F* and *wfs1a-R*, followed by Sanger sequencing. A 4 bp deletion in exon 6 was identified, resulting in a frameshift mutation predicted to produce a truncated, non-functional Wfs1a protein with loss of the transmembrane domain. To reduce potential off-target effects, F1 fish were outcrossed to WT/AB fish for at least two generations before establishing the homozygous line. Afterwards, homozygous *wfs1a* mutant zebrafish were produced through hybridization. Finally, the established homozygous *wfs1a^−/−^* mutant line was crossed with a previously established and published *wfs1b^−/−^* mutant zebrafish line [42] to generate *wfs1* knockout (*wfs1^−/−^*) zebrafish.

### 2.3. Free-Swimming Behavior Analysis

To investigate the effect of gene knockout on the motor function of zebrafish, this study conducted a free-swimming behavioral assay. On day 5 post-fertilization (5 dpf), a total of 12 zebrafish larvae per group were placed in a 24-well plate (Sangon Biotech, Shanghai, China) and acclimated under standard rearing conditions at 28.5 °C with 2 h of light adaptation. Larvae were randomly allocated to experimental groups. Behavioral scoring and data analysis were performed by an investigator blinded to genotype. Free-swimming trajectories of the larvae were recorded continuously for one hour using the Viewpoint (Saint-Cloud, France) behavioral tracking system, which was housed in an incubation chamber(Saint-Cloud, France) maintained at a constant temperature of 28.5 °C. The swimming behavior data were acquired and analyzed using the Zebralab 3.11 software (Viewpoint, Saint-Cloud, France), and the total distance moved was calculated to evaluate alterations in motor function. A sample size of 12 larvae per group was selected based on previous studies using similar behavioral paradigms in zebrafish larvae [26,27], which consistently demonstrated sufficient statistical power to detect locomotor differences in WS-related mutants.

### 2.4. In Vivo Imaging of Dorsal Distribution

The Tg (*olig2*: EGFP) strain was crossed with the generated *wfs1^−/−^* line to produce Tg (*olig2*: EGFP); *wfs1*^−/−^ zebrafish. Larvae at 3 to 5 days post-fertilization (dpf) were anesthetized with MS222 (168 mg/L, Sigma-Aldrich, St. Louis, MO, USA, E10521; pH 7.0), placed dorsal side up, and embedded in 1% low-melting agarose (Sangon Biotech, Shanghai, China). EGFP^+^ OPCs adjacent to the Mauthner cell axons were imaged in a fixed anatomical window of 800 μm × 100 μm centered directly dorsal to the cloaca, using a FV1000 confocal microscope (Olympus, Tokyo, Japan) and a water dipping lens (40×, 0.85 numerical-aperture objective, Olympus, Tokyo, Japan). Excitation was set at 488 nm, and Z-stack images were captured at a resolution of 1024 × 1024 pixels (slice thickness: 2–3 μm) [43]. In vivo imaging of dorsal distribution and myelination were conducted as previously described [44]. Using maximum intensity projection images, the number of dorsally distributed EGFP^+^ OPCs within the target anatomical region was quantified using ImageJ software (version 1.53q) [45]. Cell counting was performed by an investigator blinded to genotype.

### 2.5. In Vivo Labeling of Oligodendrocytes for Myelination

The labeling system includes two plasmids, CMV-Gal4 (50 ng/µL) and UAS-mbp-EGFP-CAAX (50 ng/µL), to specifically and randomly label the larval oligodendrocytes. Equal volumes of the two plasmids were mixed on the day of injection. Approximately 1 nL of the plasmid mixture was microinjected into the yolk of single-cell-stage embryos of wild-type and *wfs1* mutant larvae. Approximately 200 embryos were injected per group. At 5 dpf, larvae were screened under a BX60 fluorescence stereomicroscope (Olympus, Tokyo, Japan); only larvae with sparse, well-isolated EGFP-positive oligodendrocytes (1–3 cells per larva, clearly separated with no signal overlap) were selected for confocal imaging. Larvae were anesthetized with 0.4% MS-222, embedded in 1% low-melting agarose, and imaged with an Olympus FV1000 confocal microscope (40× objective) acquiring Z-stacks at 2 µm intervals. For each selected oligodendrocyte, all EGFP^+^ myelin segments extending from that cell body were counted in the maximum intensity projection. Each counted cell was derived from a different larva.

### 2.6. Quantitative Real-Time PCR (qRT-PCR)

Total RNA was extracted from the whole larvae (30 larvae per biological replicate) using RNAiso (Takara, Beijing, China, Cat No. 9108), and approximately 1 μg of RNA was reverse-transcribed into cDNA using HiScript II qRT SuperMix II (Vazyme, Nanjing, China, Cat No. R222-01). qRT-PCR was performed in a total volume of 10 μL containing 5 μL ChamQ Universal SYBR Green qPCR Master Mix and 1 μL cDNA template on a real-time quantification system (LightCycler 96, Roche, Basel, Switzerland). qPCR cycling conditions: 95 °C 30 s initial denaturation; 40 cycles of 95 °C 10 s/60 °C 30 s; melt curve analysis 65–95 °C. The mRNA expression levels were analyzed using the comparative Ct relative quantification method formula 2^−ΔΔCT^, with housekeeping gene *β-actin* mRNA used as invariant control to normalize the mRNA of target genes. Each experiment included three independent biological replicates, each consisting of pooled RNA from 30 larvae. Three technical replicates were run for each biological replicate. Primer efficiencies (95–105%) were validated by standard curve analysis. All primers used are listed in Supplementary Table S1.

### 2.7. Western Blot

Zebrafish larvae at 4 and 5 dpf (60 larvae per biological replicate) were collected and homogenized on ice in RIPA lysis buffer (Servicebio, Wuhan, China, Cat No. G2002) supplemented with protease inhibitors (Beyotime, Shanghai, China). The lysates were centrifuged at 12,000× *g* for 15 min at 4 °C, and the supernatants were collected. Protein concentration was determined using a BCA assay kit (Beyotime, Shanghai, China, Cat No. P0009). Protein samples were diluted with 5× SDS loading buffer (Beyotime, Shanghai, China), separated by 10% SDS-PAGE (prepared in-house from reagents purchased from Sangon Biotech, Shanghai, China) and then transferred onto PVDF membranes (Millipore, Burlington, MA, USA) that were pre-activated in methanol (Sigma-Aldrich, St. Louis, MO, USA). The membranes were blocked with 5% skim milk in TBST (Sangon Biotech, Shanghai, China) at room temperature for 1 h, followed by incubation with primary antibodies diluted in blocking buffer at 4 °C overnight. The primary antibodies used were: rabbit anti-Mbp (1:1000, Boster, Wuhan, China, Cat No. BA0094, RRID: AB_2665438), rabbit anti-Hmgcs1 (1:1000, Proteintech, Rosemont, IL, USA, Cat No. 17643-1-AP, RRID: AB_2248359), and mouse anti-Gapdh (1:2000, HuaAn, Shanghai, China, Cat No. R1108-1, RRID: not available). After washing with TBST, the membranes were incubated with HRP-conjugated goat anti-rabbit or anti-mouse secondary antibodies (1:5000, Proteintech, Rosemont, IL, USA) at room temperature for 1 h. Protein signals were detected using an ECL chemiluminescence substrate (Thermo Fisher, Waltham, MA, USA, Cat No. 34577) and captured with a GE ImageQuant LAS 4000 luminescent image analyzer (GE Healthcare, Chicago, IL, USA). Images were acquired using incremental exposure to ensure non-saturated signals, and representative exposures were selected for presentation. The band intensities were quantified using ImageJ software and normalized to the intensity of Gapdh. Full uncropped blot images have been provided to the editorial office as separate files.

### 2.8. Transmission Electron Microscopy (TEM)

MS-222 working solution and glutaraldehyde fixative were freshly prepared using phosphate-buffered saline (PBS, Sangon Biotech, Shanghai, China)). Larvae were anesthetized with MS-222 and subsequently decapitated using fine surgical scissors. The heads were immediately transferred with precision forceps into 2.5% glutaraldehyde (Sigma-Aldrich, St. Louis, MO, USA) fixative for rapid fixation. Samples were fixed overnight at room temperature on a shaker, followed by three washes with 0.1 M cacodylate buffer (Sigma-Aldrich, St. Louis, MO, USA) (5 min each). Post-fixation was performed in 1% osmium tetroxide (Sigma-Aldrich, St. Louis, MO, USA) for 2 h at room temperature, after which samples were rinsed once with ddH_2_O. Dehydration was initiated with a 15 min incubation in 50% ethanol (Sinopharm, Shanghai, China), followed by en bloc staining in 70% uranyl acetate ethanol solution (Sigma-Aldrich, St. Louis, MO, USA) overnight on a shaker. Gradual dehydration continued with 80%, 95%, and 100% ethanol (15 min each). Samples were infiltrated using a propylene oxide (Sigma-Aldrich, St. Louis, MO, USA) /epoxy resin (Sigma-Aldrich, St. Louis, MO, USA) gradient (1 h per step) and embedded in pure epoxy resin (Sigma-Aldrich, St. Louis, MO, USA). After orientation with pointed forceps, blocks were cured at 65 °C overnight. Cured blocks were trimmed with a razor blade, and ultrathin sections (70–90 nm) were cut using an LKB NOVA ultramicrotome (Lecia, Berlin, Germany). Sections were mounted on copper grids and imaged with a JEM-1230 electron microscope (JEOL, Tokyo, Japan). For each group, 6 larvae were analyzed. From each larva, 1 ultrathin cross-section through the hindbrain at the level of the Mauthner cell axon was examined. Per section, 1 Mauthner axon profile was identified based on their characteristic large diameter and position, and myelin thickness was quantified. TEM image analysis and myelin thickness measurements were performed by an investigator blinded to genotype. Residual blocks were stored in 2 mL centrifuge tubes within a desiccator. Negatives were digitized using a scanner (Epson, Suwa, Japan) and analyzed with ImageJ software.

### 2.9. Drug Treatment

Geranylgeraniol (GGOH, Sangon Biotech, Shanghai, China, Cat No. A411208-0200) was dissolved in dimethyl sulfoxide (DMSO, Sangon Biotech, Shanghai, China) to prepare a 1 M work solution. For experimental treatment, 1 nL of GGOH solution (1 M, yielding an estimated final concentration of approximately 0.1–0.5 mM in the yolk based on yolk volume estimates) [30] or an equal volume of DMSO (vehicle control, final estimated DMSO concentration < 0.1%) was microinjected into the yolk of zebrafish embryos at 24 hpf. The injection volume and developmental stage were selected based on established protocols for compound delivery in zebrafish embryos.

### 2.10. Fluorescence-Activated Cell Sorting (FACS)

Larvae at 5 dpf (300 per group) were collected and dissociated in ice-cold HBSS buffer (Sangon Biotech, Shanghai, China). Single-cell suspensions were prepared by enzymatic digestion with trypsin-EDTA solution (Sangon Biotech, Shanghai, China, Cat No. E607002) at 37 °C for 20 min with gentle pipetting every 5 min to promote dissociation and mechanical disruption. Digestion was terminated by addition of fetal bovine serum (Gibco, Thermo Fisher Scientific, Waltham, MA, USA). The cell suspensions were sequentially filtered through a 100 µm and then a 40 µm cell strainer (Sangon Biotech, Shanghai, China) to obtain a single-cell suspension [46]. EGFP^+^ cells were gated based on fluorescence intensity compared to non-transgenic wild-type larvae used as negative gating controls. The purity of sorted olig2^+^ cells was confirmed by post-sort reanalysis, which showed >90% EGFP positivity. The Tg(*olig2*: EGFP) transgenic line has been extensively validated as a specific marker of OPCs and oligodendrocytes in previous zebrafish studies [38,47,48]; however, sorted cells were not further validated by independent markers such as sox10. Samples were analyzed via flow cytometry (BD FACS Aria III, Franklin Lakes, NJ, USA) and divided into EGFP^+^ cells and EGFP^−^ cells. Total RNA was extracted using TRIzol Reagent (Invitrogen, Waltham, MA, USA) for subsequent qRT-PCR analysis.

### 2.11. RNA Sequencing

Total RNA was extracted from 5 dpf *wfs1^+/+^* and *wfs1^−^^/^^−^* larvae (30 larvae per biological replicate, three biological replicates per group) using the same protocol as described in Section 2.6. RNA quality was assessed by NanoDrop spectrophotometry (OD_260_/_280_ ratio 1.8–2.1, Thermo Fisher Scientific, Waltham, MA, USA)) and Agilent Bioanalyzer (RIN ≥ 8.0, Agilent Technologies, Santa Clara, CA, USA)). Library preparation was performed using poly(A) selection to enrich mRNA, followed by fragmentation, reverse transcription, and paired-end library construction. Sequencing was performed on an Illumina NovaSeq 6000 platform (Illumina, Inc., San Diego, CA, USA) with 150 bp paired-end reads, generating a minimum of 20 million read pairs per sample. Raw reads were quality-filtered using fastp (v0.18.0). Reads were aligned to the zebrafish reference genome (GRCz11, Ensembl release 109) using HISAT2 (v2.2.8). Gene expression was quantified using RSEM (v1.3.3) and normalized to transcripts per million (TPM). Differential expression analysis was performed using DESeq2 (v1.34.0), with differentially expressed genes (DEGs) defined as adjusted *p* value (FDR) < 0.05 and |log_2_ fold change| >0.585 (corresponding to >1.5-fold change). GO and KEGG pathway enrichment analyses were performed using OmicShare Tools (https://www.omicshare.com/tools).

### 2.12. TUNEL Staining

Apoptotic cells were detected using the TUNEL BrightRed Apoptosis Detection Kit A113 (Vazyme, Nanjing, China, Cat No. A113-01) according to the manufacturer’s instructions with minor modifications for zebrafish larvae. Briefly, 3 dpf and 5 dpf larvae were fixed in 4% paraformaldehyde (PFA) in PBS overnight at 4 °C, followed by dehydration in a graded methanol series and rehydration. Larvae were permeabilized with proteinase K (10 μg/mL in PBS) for 10 min at room temperature, re-fixed in 4% PFA for 20 min, and washed with PBS. TUNEL reaction mixture was prepared by diluting TdT enzyme in labeling buffer as directed, applied to samples, and incubated at 37 °C for 60 min in the dark. Samples were washed three times with PBS and mounted in 1% low-melting agarose. Fluorescence imaging was performed on Olympus FV1000 confocal microscope (40× objective) acquiring Z-stacks at 2 µm intervals. TUNEL^+^ cells were quantified within the fixed anatomical window of 800 μm × 100 μm centered directly dorsal to the cloaca. A total of 13 larvae per genotype were analyzed.

### 2.13. Statistical Analysis

Graphs and statistical significance were analyzed with GraphPad Prism 9.0 software (San Diego, CA, USA), Adobe Photoshop CC2020 (Adobe Systems, Mountain View, CA, USA), Adobe Illustrator CC 2020 (Adobe Systems, Mountain View, CA, USA) and Figdraw (Home for Researchers, Hangzhou, China). Data were presented as mean ± standard error of the mean (SEM). For qRT-PCR experiments involving multiple genes at a single timepoint, unpaired two-tailed Student’s *t*-tests were applied for each gene, with Bonferroni correction applied across all 5 comparisons. *p* values reported are Bonferroni-adjusted (raw *p* values multiplied by 5 for 5 comparisons). Comparisons between two groups at a single timepoint with a single outcome measure were conducted using unpaired two-tailed Student’s *t*-tests without correction. For qRT-PCR data of myelin marker genes measured across multiple timepoints, two-way ANOVA was performed with genotype and developmental timepoint as independent variables, followed by Sidak’s post hoc test for pairwise comparisons at each timepoint. For comparisons among more than two groups, one-way analysis of variance (ANOVA) was applied, followed by Tukey’s post hoc test. For experimental designs involving two independent variables, two-way ANOVA was used, with Sidak’s or Tukey’s post hoc test as appropriate. Statistical significance was defined as *p < 0.05, **p < 0.01, and ***p < 0.001, ****p < 0.0001. All experiments were repeated at least three times. Detailed sample sizes and specific statistical tests were provided in the corresponding Figure legends.

## 3. Results

### 3.1. Generation of wfs1^−/−^ Zebrafish

**Figure 1:**
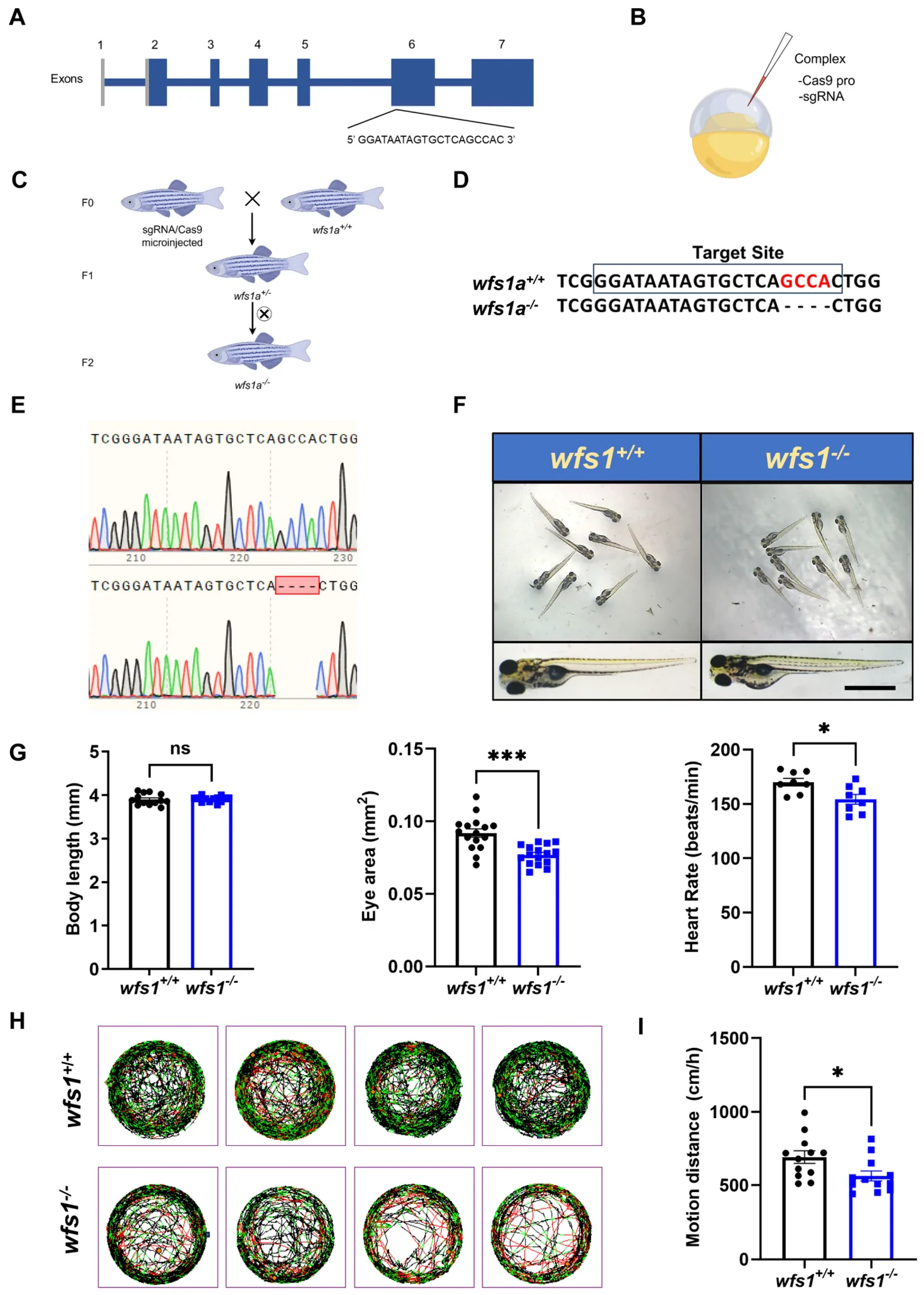
**Generation of *wfs1^−/−^* zebrafish.** (**A**) CRISPR-Cas9 targeting strategy for *wfs1a* exon 6. Arrow indicates sgRNA-binding site. (**B**) Microinjection schematic at one-cell stage. Cas9 protein and sgRNA were co-injected into single-cell embryos. (**C**) Hybridization of *wfs1*^−/−^ transgenic line: *wfs1a*^−/−^ and *wfs1b*^−/−^ zebrafish were crossed for two consecutive generations to obtain *wfs1*^−/−^ lines. (**D**) Target sequence in the sixth exon of *wfs1a*. (**E**) Sanger sequencing chromatogram. A 4 bp deletion (dashed box) in exon 6 caused a frameshift mutation. (**F**) Representative images of 5 dpf *wfs1^+/+^* and *wfs1^−/−^* larvae. Scale bar: 1 mm. (**G**) Quantification of body length, eye size, and heart rate in 5 dpf larvae. Data represent mean ± SEM. Body length: n = 13 biologically independent animals/group; eye area: n = 16 biologically independent animals/group; heart rate: n = 8 biologically independent animals/group. ns: not significant, ns, not significant, * *p* < 0.05, *** *p* < 0.001, assessed by unpaired two-tailed Student’s *t*-test. (**H**) Heatmaps of spontaneous movement trajectories. Red: high activity; green: low activity. (**I**) Quantification of total movement distance. *wfs1*^−/−^ larvae showed reduced motility. Data represent mean ± SEM and n = 12 biologically independent animals/group. * *p* < 0.05, assessed by unpaired two-tailed Student’s *t*-test.

Given the presence of two *wfs1* paralogs (*wfs1a* and *wfs1b*) in zebrafish [26] and the preexisting *wfs1b^−/−^* homozygous line in our laboratory [42], we generated a *wfs1a* mutant (*wfs1a^−/−^*). A target site was designed in the sixth exon of *wfs1a* (Figure 1A), and sgRNA with recombinant Cas9 protein was microinjected into the yolk of one-cell-stage embryos (Figure 1B). After two generations of outcrossing and genotyping, we obtained homozygous *wfs1a^−/−^* mutants (Figure 1C). Sanger sequencing confirmed a 4 bp deletion in the sixth exon, resulting in a frameshift mutation (Figure 1D,E). *wfs1a^−/−^* zebrafish were then crossed with the established *wfs1b^−/−^* line. Through successive crosses and genotypic validation, we established a stable *wfs1* knockout line.

To assess pathological consequences of *wfs1* deficiency, we analyzed systemic phenotypes in 5 dpf *wfs1^−/−^* larvae. Morphometric analysis revealed no significant difference in body length relative to WT/AB controls; however, *wfs1^−/−^* larvae exhibited significantly reduced eye area (Supplementary Figure S1) and heart rate (Figure 1F,G). Heart rate was measured under a stereomicroscope by direct visual counting of cardiac contractions over a 30 s period (converted to beats per minute) from video recordings. Representative images of the reduced eye area are shown in Supplementary Figure S1. Spontaneous locomotor activity was quantified by tracking total movement distance over 1 h. *wfs1*^−/−^ larvae showed a 47.8% reduction in cumulative movement distance compared to WT (Figure 1H,I), indicating impaired spontaneous motility. Quantification of body length, eye area, heart rate, and movement distance was performed by an investigator blinded to genotype. Animals were randomly allocated to experimental groups. These results further indicated that we successfully established a homozygous *wfs1* mutant zebrafish line. Moreover, these phenotypes are broadly consistent with mammalian models of WS, indicating the utility of *wfs1^−/−^* zebrafish for studying WS-associated neurodevelopmental pathology.

### 3.2. wfs1 Deficiency Delayed Dorsal Distribution of OPCs and Hindered Myelination of OLs

**Figure 2:**
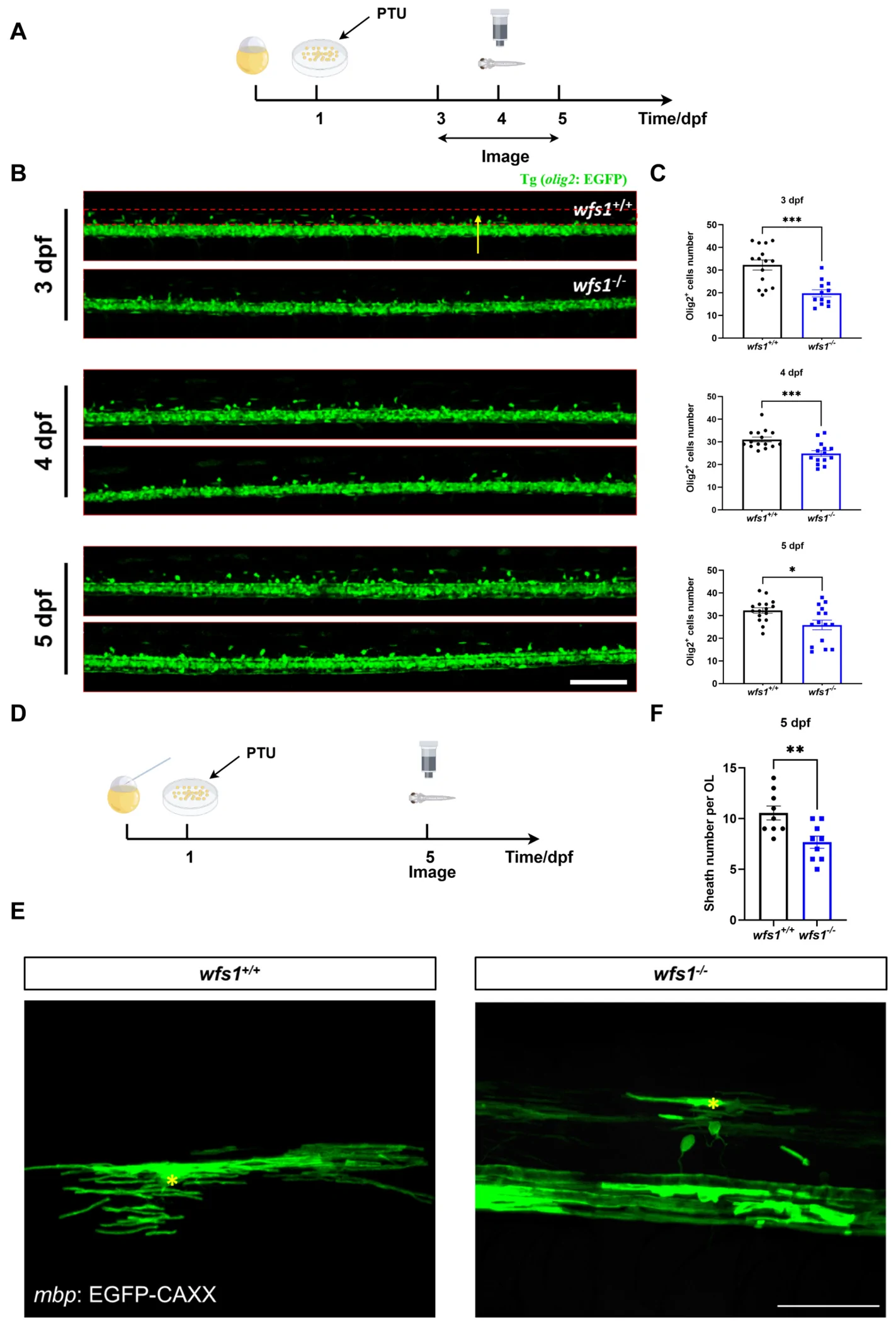
***wfs1^−/−^* delayed dorsal distribution of OPCs and hindered myelination capacity.** (**A**) Experimental timeline for in vivo dorsal distribution tracking. (**B**) Representative image of dorsally migrated *olig2^+^* cells at 3, 4, and 5 dpf between *wfs1^+/+^* and *wfs1^−/−^* groups. EGFP^+^ OPCs adjacent to the Mauthner cell axons were imaged in a fixed anatomical window of 800 μm × 100 μm centered directly dorsal to the cloaca. Arrows indicate the direction of *olig2^+^* cell migration from ventral to dorsal, and the boxed areas mark the regions used for quantification. Scale bar: 20 μm. (**C**) The number of dorsally distributed *olig2^+^* cells at 3, 4, and 5 dpf decreased in *wfs1^−/−^* group. Data represent mean ± SEM and n ≥ 13 biologically independent animals/group. * *p* < 0.05, *** *p* < 0.001, assessed by unpaired two-tailed Student’s *t*-test. (**D**) Experimental workflow for in vivo OL myelination. (**E**) Typical imaging of individual oligodendrocytes labeled with plasmid CMV-Gal4 and UAS-mbp-EGFP-CAAX in the *wfs1^+/+^* and *wfs1^−/−^* groups. The yellow asterisks denote the positions of oligodendrocyte cell bodies. Scale bar: 50 μm. (**F**) Number of individual oligodendrocyte-wrapped myelin segments in the *wfs1^+/+^* and *wfs1^−/−^* groups. Wild type: n = 9 derived from 9 larvae; *wfs1* mutant: n = 9 derived from 9 larvae. Data represent mean ± SEM. ** *p* < 0.01, assessed by unpaired Student’s *t*-test.

Given that Mauthner neuronal axons are among the first to be myelinated during development, we first observed the OPC dorsal distribution of the M-cell in *wfs1*^−/−^ larvae to investigate the direct impact of *wfs1* deficiency on the development of oligodendrocyte and myelin structure. The *wfs1^−/−^* zebrafish were crossed with the Tg (*olig2*: EGFP) transgenic line (labeling the oligodendrocyte lineage) to generate Tg (*olig2*: EGFP); *wfs1^−/−^* zebrafish. Using in vivo confocal imaging, we tracked the distribution of OPCs adjacent to the M-cell axon in both control and *wfs1^−/−^* zebrafish larvae from 3 dpf to 5 dpf (Figure 2A). Quantitative analysis revealed a significant reduction in the number of dorsally distributed OPCs in the *wfs1^−/−^* group (Figure 2B,C), indicating that *wfs1* deficiency specifically reduces the dorsal distribution of OPCs along the M-cell axon. Whether this reflects impaired directional migration, reduced OPC proliferation or survival, or a combination of these processes requires further investigation with dynamic cell tracking or proliferation/apoptosis markers.

To further investigate the effect on the myelination ability of *wfs1* mutant, we randomly labeled oligodendrocytes with plasmid UAS-mbp-EGFP-CAAX and observed the number of single oligodendrocyte-wrapped myelin segments in the *wfs1^−/−^* group (Figure 2D). The imaging results indicated that the number of myelin segments wrapped by each oligodendrocyte in *wfs1^−/−^* larvae was significantly lower than that of the *wfs1^+/+^* group (Figure 2E,F). For statistical analysis of myelin segment data, the larva was treated as the biological unit to avoid pseudoreplication.

To investigate whether the reduced dorsal distribution of OPCs in *wfs1^−^^/^^−^* larvae results from increased apoptosis, we performed TUNEL staining in Tg(*olig2*: EGFP); *wfs1^−^^/^^−^* larvae and their wild-type siblings at both 3 dpf and 5 dpf (Supplementary Figure S2). TUNEL^+^ cells were detected at low and comparable levels in both genotypes within the fixed anatomical window above the cloaca at both time points. Importantly, the number of TUNEL^+^/EGFP^+^ double-positive cells remained very low, with almost no co-labeled cells detected per larva in either genotype at either developmental stage. This is consistent with previous observations demonstrating that zebrafish OPCs largely survive pathological insults and rarely undergo cell death [49]. Taken together, these data demonstrate that the reduced OPC dorsal distribution in *wfs1^−^^/^^−^* larvae is not attributable to enhanced apoptosis, suggesting that impaired dorsal distribution may instead underlie this phenotype.

### 3.3. wfs1 Deficiency Caused Early Hypomyelination of Mauthner Axons and Downregulated Myelin Marker Genes Expression

**Figure 3:**
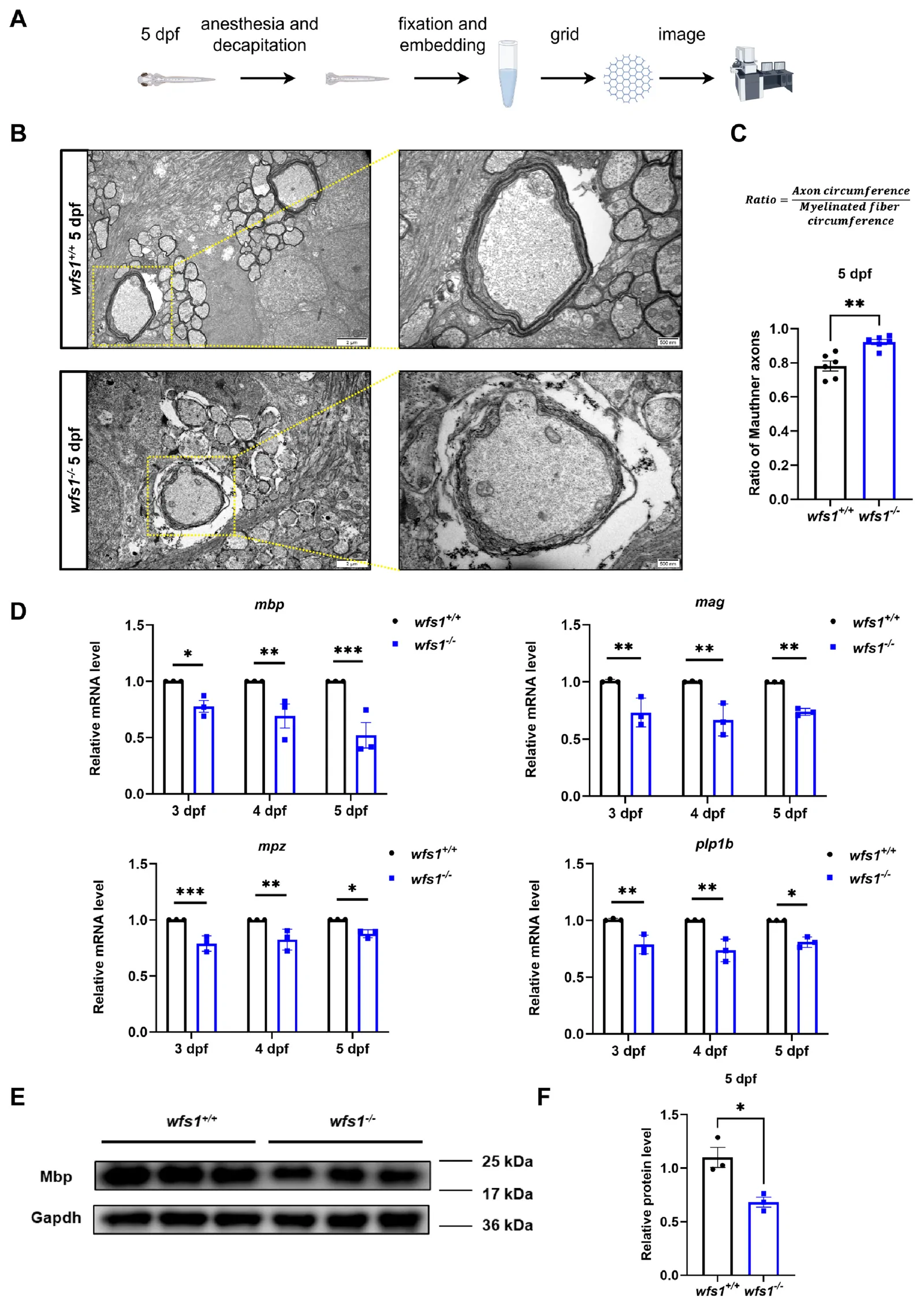
***wfs1^−/−^* suppressed myelination in zebrafish.** (**A**) Experimental workflow for sample preparation and transmission electron microscopy observation of Mauthner cell axonal myelination in 5 dpf zebrafish larvae. (**B**) Transmission electron micrographs of the axons of Mauthner cells in *wfs1^+/+^* and *wfs1^−/−^* groups of 5 dpf zebrafish larvae. Scale bars: 2 μm (left), 500 nm (right). (**C**) Myelin thickness analysis of Mauthner cells in *wfs1^+/+^* and *wfs1^−/−^* groups. Data represent mean ± SEM and n = 6 biologically independent animals/group. ** *p* < 0.01, assessed by unpaired two-tailed Student’s *t*-test. (**D**) qRT-PCR analysis of myelin marker genes. Relative mRNA expression of *mbp*, *mag*, *mpz*, and *plp1b* at 3, 4, and 5 dpf decreased in *wfs1^−/−^* group. All mRNA expression values were normalized to the housekeeping gene *β-actin*. Data represent mean ± SEM; Each experiment included three biologically independent samples. Each sample contained pooled RNA from 30 larvae. Three technical replicates were run for each sample. Statistical analysis was performed using two-way ANOVA (genotype × timepoint) followed by Sidak’s multiple comparisons test. Main effect of genotype: *mag*, F(1,12) = 32.53, *p* < 0.0001; *mbp*, F(1,12) = 42.68, *p* < 0.0001; *mpz*, F(1,12) = 52.07, *p* < 0.0001; *plp1b*, F(1,12) = 40.15, *p* < 0.0001. Main effect of timepoint and interaction were not significant for any gene (*p* > 0.05). Post hoc comparisons (Sidak’s test): *mbp*: 3 dpf * *p* = 0.0238, 4 dpf ** *p* = 0.0039, 5 dpf *** *p* = 0.0001; *mag*: 3 dpf ** *p* = 0.0042, 4 dpf ** *p* = 0.0014, 5 dpf ** *p* = 0.0086; *mpz*: 3 dpf *** *p* = 0.0007, 4 dpf ** *p* = 0.0027, 5 dpf * *p* = 0.0203; *plp1b*: 3 dpf ** *p* = 0.0054, 4 dpf ** *p* = 0.0017, 5 dpf * *p* = 0.0137. (**E**) Representative Western blot results of Mbp protein between *wfs1^+/+^* and *wfs1^−/−^* groups. Gapdh served as the loading control; lanes 1–3: *wfs1^+/+^*; lanes 4–6: *wfs1^−/−^*. (**F**) Western blot analysis showed that Mbp protein expression is inhibited in the *wfs1^−/−^* group compared with that of the *wfs1^+/+^* group. The protein expressions were quantified by ImageJ software. Each experiment included three biologically independent samples. Each sample contained 60 larvae. Data represent mean ± SEM. * *p* < 0.05, assessed by unpaired two-tailed Student’s *t*-test. The original Western blot images are summarized in File S2.

To further elucidate the pathological consequences of the reduced OPC dorsal distribution on myelination, we performed ultrastructural analysis of Mauthner axons at 5 dpf using transmission electron microscopy (TEM) (Figure 3A). We used the ratio of the inner myelin perimeter to the outer myelin perimeter as a metric for evaluation. A higher value of this ratio indicates thinner myelin. TEM results showed significantly thinner myelin and signs of hypomyelination in *wfs1^−^^/^^−^* larvae compared to *wfs1^+/+^* controls, indicating early hypomyelination (Figure 3B,C).

To determine whether this hypomyelination phenotype persists at later developmental stages, we further performed TEM analysis at 8 dpf. Interestingly, the myelin thickness of M-cell axons showed no significant difference between *wfs1^−/−^* and *wfs1^+/+^* siblings at this stage (Supplementary Figure S3), suggesting partial developmental compensation in this axonal population by 8 dpf. The mechanism underlying this recovery remains to be determined; possible explanations include compensatory upregulation of alternative isoprenoid synthesis enzymes, delayed but eventually complete OPC maturation, or activation of other metabolic pathways. It should be noted, however, that these observations are based on discrete sampling of the Mauthner axon and may not reflect global myelination status across all axonal populations or brain regions. Furthermore, given the inherent interspecies differences between zebrafish and humans, caution is warranted in direct extrapolation: in human Wolfram syndrome patients, myelin abnormalities manifest as developmental hypomyelination that has been reported across multiple white matter tracts [2,12], a discrepancy that may reflect divergent compensatory capacities between species. These findings indicate that the loss of *wfs1* exerts its most pronounced impact on myelination during early developmental stages, which may represent a tractable window for therapeutic intervention.

Furthermore, we sought to investigate the underlying molecular mechanisms by examining the expression levels of key markers involved in myelin development. We first detected the expression of the classic myelin-related genes *mbp*, *mag*, *mpz*, and *plp1b* in *wfs1* mutant zebrafish at 3, 4, and 5 dpf by qRT-PCR. Two-way ANOVA revealed a highly significant main effect of genotype on the expression of all four myelin genes, confirming that *wfs1* deficiency leads to broad transcriptional suppression of core myelin genes across developmental stages (Figure 3D). Sidak’s post hoc comparisons demonstrated significant downregulation at each individual timepoint (Figure 3D). Whole-larvae qRT-PCR may be influenced by systemic developmental changes; the more definitive cell-type-specific evidence from FACS-sorted olig2^+^ cells is presented in Section 3.5. Moreover, we further investigated the expression of protein levels through Western blot experiments. Analysis results showed that *wfs1* mutation significantly reduced the expression of Mbp protein (Figure 3E,F), which is highly consistent with the qRT-PCR results. In conclusion, both imaging and molecular results indicated that the *wfs1* mutation causes early hypomyelination of M-cell axons at 5 dpf.

### 3.4. Transcriptomic Profiling Suggested wfs1 Deficiency Impairs OPCs Development by Suppressing the hmgcs1-Mediated Isoprenylation Pathway

**Figure 4:**
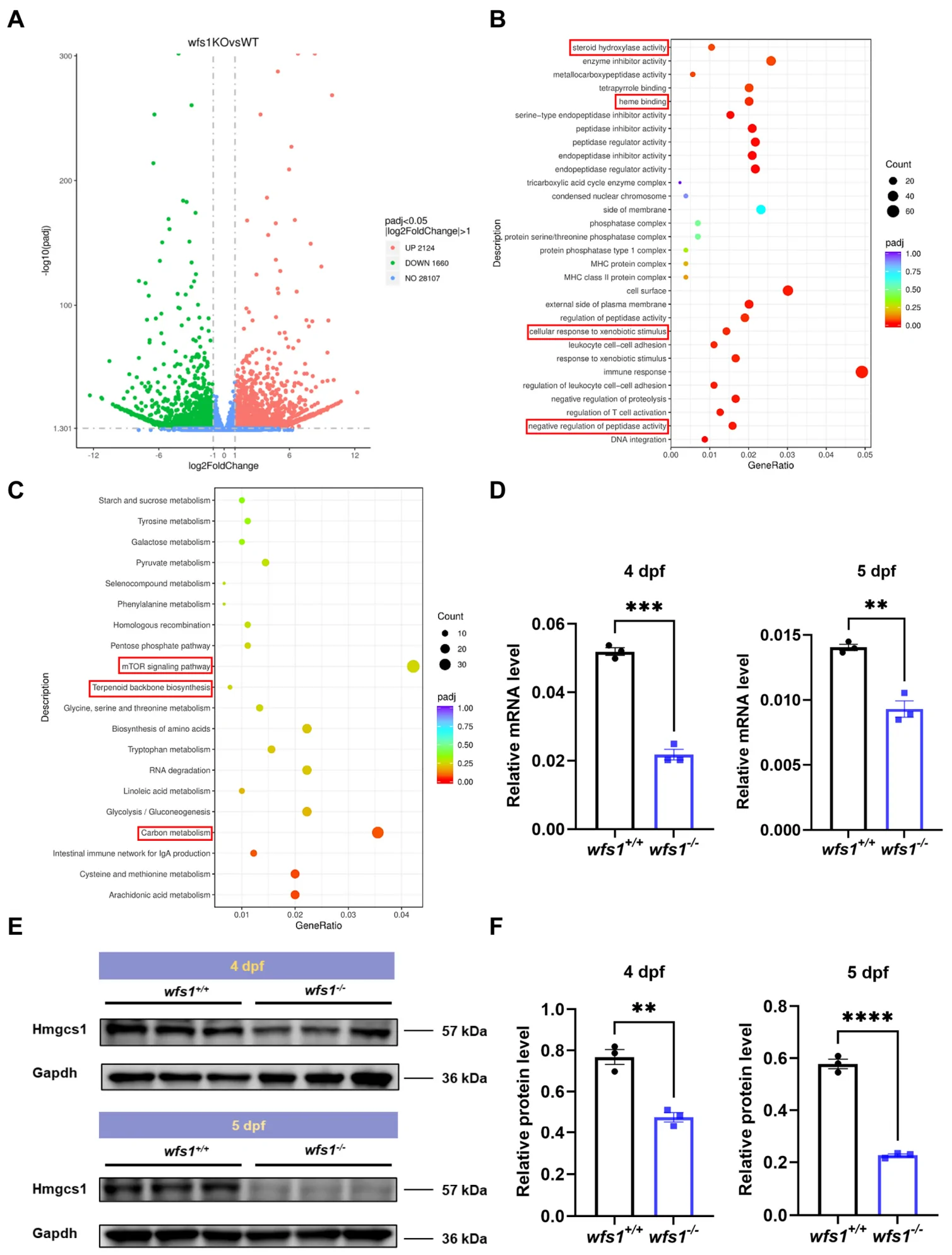
***wfs1^−/−^* downregulated *hmgcs1* expression**. (**A**) Analysis of differentially expressed genes between *wfs1^+/+^* and *wfs1^−/−^* groups. Volcano plot showing each gene plotted according to its log2 fold change. All highly differentially expressed genes with adjusted *p* (FDR) < 0.05 are in red with fold change > 1.5. In total, 2124 genes were significantly upregulated and 1660 genes were significantly downregulated. (**B**) Bubble plot of GO enrichment. Dot size represents the number of genes; color intensity indicates statistical significance (–log_10_(p)). The red boxes highlight the functional terms that are the main focus of subsequent mechanistic validation. (**C**) Heatmap of KEGG pathway enrichment. Red: upregulated pathways; purple: downregulated pathways. The red boxes highlight the functional pathways that are the main focus of subsequent mechanistic validation. (**D**) qRT-PCR analysis of *hmgcs1*. Relative mRNA expression of *hmgcs1* at 4 and 5 dpf decreased in *wfs1^−/−^* group. All mRNA expression values were normalized to the housekeeping gene *β-actin*. Data represent mean ± SEM; Each experiment included three biologically independent samples. Each sample contained pooled RNA from 30 larvae. Three technical replicates were run for each sample. ** *p* < 0.01, *** *p* < 0.001, assessed by unpaired two-tailed Student’s *t*-test. (**E**) Representative Western blot results of Hmgcs1 protein at 4 and 5 dpf between *wfs1^+/+^* and *wfs1^−/−^* groups. Gapdh served as the loading control; lanes 1–3: *wfs1^+/+^*; lanes 4–6: *wfs1^−/−^*. (**F**) Western blot analysis showed that Hmgcs1 protein expression is inhibited in the *wfs1^−/−^* group compared with that of the *wfs1^+/+^* group. The protein expressions were quantified by ImageJ software. Each experiment included three biologically independent samples. Each sample contained 60 larvae. Data represent mean ± SEM. ** *p* < 0.01, **** *p* < 0.0001, assessed by unpaired two-tailed Student’s *t*-test. The original Western blot images are summarized in File S2.

Given the observed delay in dorsal distribution of OPCs and broad suppression of myelin gene expression in *wfs1^−/−^* zebrafish, we performed transcriptome sequencing to elucidate the upstream molecular mechanisms underlying these phenotypes (File S1). Transcriptome analysis revealed a total of 3784 differentially expressed genes (DEGs) between *wfs1^+/+^* and *wfs1^−/−^* groups, with 2124 genes significantly upregulated and 1660 genes significantly downregulated (Figure 4A). DEGs were defined using adjusted *p* value (FDR) < 0.05 and fold change > 1.5. GO (Gene Ontology) term analysis revealed significant alterations in genes associated with steroid hydroxylase activity, proteolysis, and the tricarboxylic acid cycle in *wfs1* mutants (Figure 4B). KEGG (Kyoto Encyclopedia of Genes and Genomes) pathway enrichment analysis further indicated that differentially expressed genes were predominantly involved in carbon metabolism, amino acid metabolism, and steroid biosynthesis pathways (Figure 4C).

Among the differentially expressed genes, we identified *hmgcs1*, which encodes 3-hydroxy-3-methylglutaryl-CoA synthase 1, an upstream enzyme that catalyzes the condensation of acetyl-CoA to HMG-CoA, providing substrate for the downstream rate-limiting enzyme HMGCR in the mevalonate pathway. This positions *hmgcs1* at the common source of two critical branches: cholesterol synthesis and the generation of farnesyl pyrophosphate (FPP) and geranylgeranyl pyrophosphate (GGPP), which serve as direct donors for protein isoprenylation. Previous studies have shown that mutation of *hmgcs1* impedes dorsal distribution toward target axons through protein isoprenylation and disrupts myelin gene expression [30,50,51]. We therefore examined the mRNA and protein expression of *hmgcs1* in both control and *wfs1^−/−^* groups at 4 and 5 dpf. The analysis results demonstrated a significant downregulation of *hmgcs1* at both mRNA and protein levels in *wfs1^−/−^* zebrafish (Figure 4D–F). These findings suggest that *wfs1* deficiency is associated with *hmgcs1* downregulation, supporting involvement of this pathway in OPC dorsal distribution and myelination defects, although direct regulation of *hmgcs1* transcription by *wfs1* has not been demonstrated.

To determine whether classical ER stress signaling is concurrently activated in *wfs1^−^^/^^−^* larvae, we examined the expression of canonical ER stress markers at 5 dpf by qRT-PCR. The results showed significant upregulation of *atf6*, *chop*, *hspa5 (bip)*, and *xbp1s* in *wfs1^−^^/^^−^* larvae compared to *wfs1^+/+^* controls (Supplementary Figure S4), confirming that *wfs1* deficiency induces ER stress in this model. These findings indicate that both ER stress activation and *hmgcs1*-dependent isoprenylation suppression co-exist in *wfs1^−/−^* zebrafish, although whether these two pathways act independently, synergistically, or in a hierarchical manner remains to be elucidated.

### 3.5. FACS Confirmed wfs1 Deficiency Specifically Suppresses the Expression of hmgcs1 and Myelin Genes in olig2^+^ Cells

**Figure 5:**
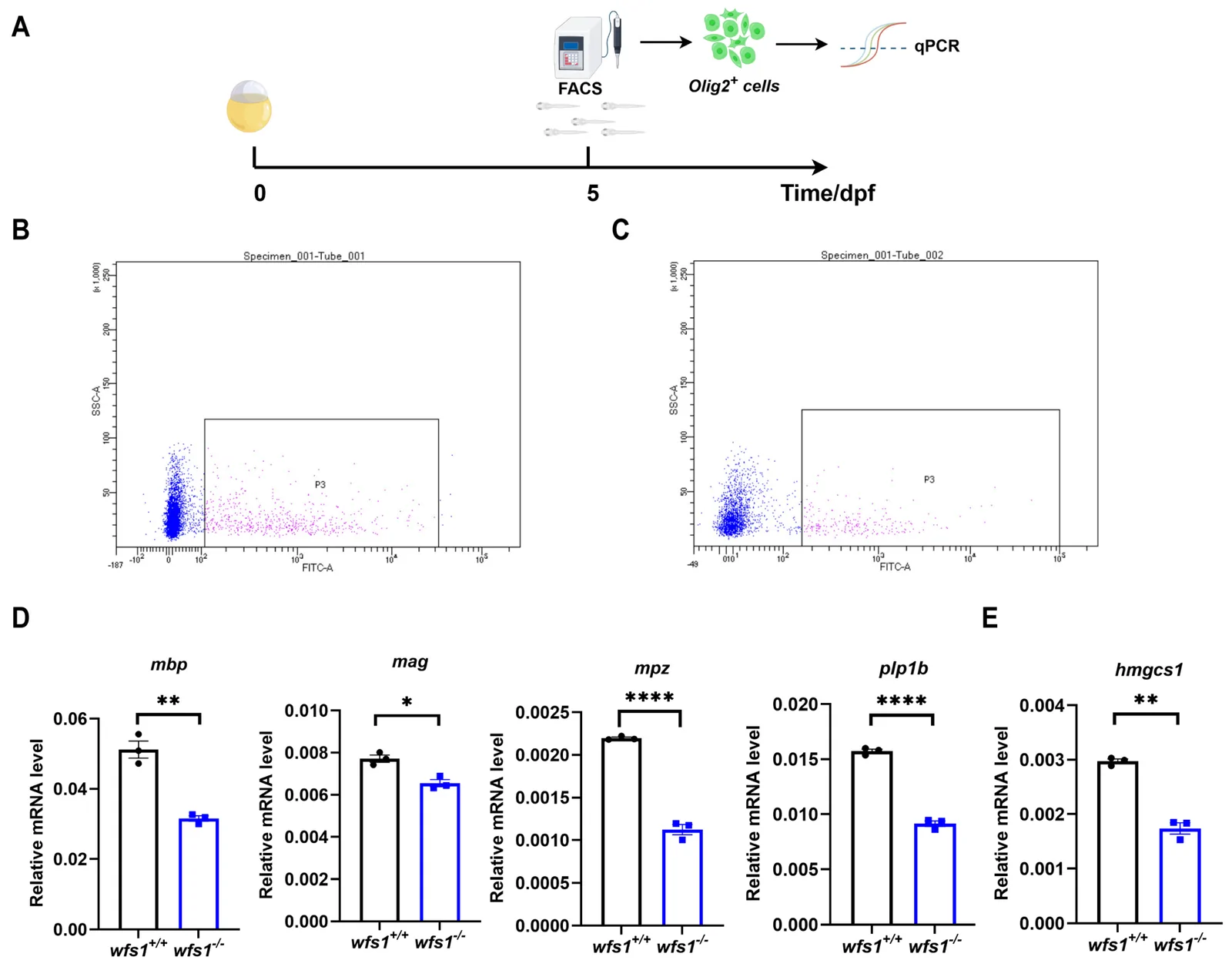
***wfs1*^−/−^ specifically suppressed myelin gene and *hmgcs1* expression in olig2^+^ cells**. (**A**) Experimental timeline shows that, at 5 dpf, *olig2^+^* labeled cells from the *wfs1*^−/−^ and *wfs1*^+/+^ groups were collected using FACS, followed by detection with qRT-PCR. (**B**) Flow cytometry analysis of sorted cell populations from Tg (*olig2*: EGFP); *wfs1^+/+^* zebrafish. FSC: forward scatter; SSC: side scatter. (**C**) Flow cytometry analysis of sorted cell populations from Tg (*olig2*: EGFP); *wfs1^−/−^* zebrafish. FSC: forward scatter; SSC: side scatter. (**D**,**E**) qRT-PCR analysis of myelin marker genes in sorted *olig2^+^* cells. Relative mRNA expression of *mbp*, *mag*, *mpz*, *plp1b* and *hmgcs1* at 5 dpf decreased in *wfs1^−/−^* group. All mRNA expression values were normalized to the housekeeping gene *β-actin*. Data represent mean ± SEM; Each experiment included three biologically independent samples. Each biological replicate consisted of *olig2^+^* cells sorted from 300 larvae. Three technical replicates were run for each sample. *p* values are Bonferroni-adjusted from individual unpaired two-tailed Student’s *t*-tests. * *p* < 0.05; ** *p* < 0.01; **** *p* < 0.0001.

To further specifically confirm the effect of *hmgcs1* downregulation on OPCs in *wfs1* mutants, we performed olig2^+^ cell-specific sorting and gene expression analysis in 5 dpf larvae (Figure 5A). We isolated *olig2^+^* cells from both control and *wfs1^−/−^* larvae at 5 dpf using FACS (Figure 5B,C), followed by qRT-PCR analysis. Post-sort reanalysis confirmed >90% purity of the EGFP^+^ population. The results demonstrated significant downregulation of *hmgcs1* in the *wfs1^−/−^* group (Figure 5E). In addition, we found that the expression of myelin-related genes in *wfs1* mutant *olig2^+^* cells was significantly downregulated (Figure 5D), providing stronger cell-type-specific evidence for the myelin development defect of the *wfs1* mutant, complementing the whole-larvae qRT-PCR data. These findings indicated that *wfs1* deficiency is associated with suppression of *hmgcs1* and myelin gene expression within OPCs.

### 3.6. Geranylgeraniol Specifically Rescued Dorsal Distribution Defects in wfs1^−/−^ Zebrafish

**Figure 6:**
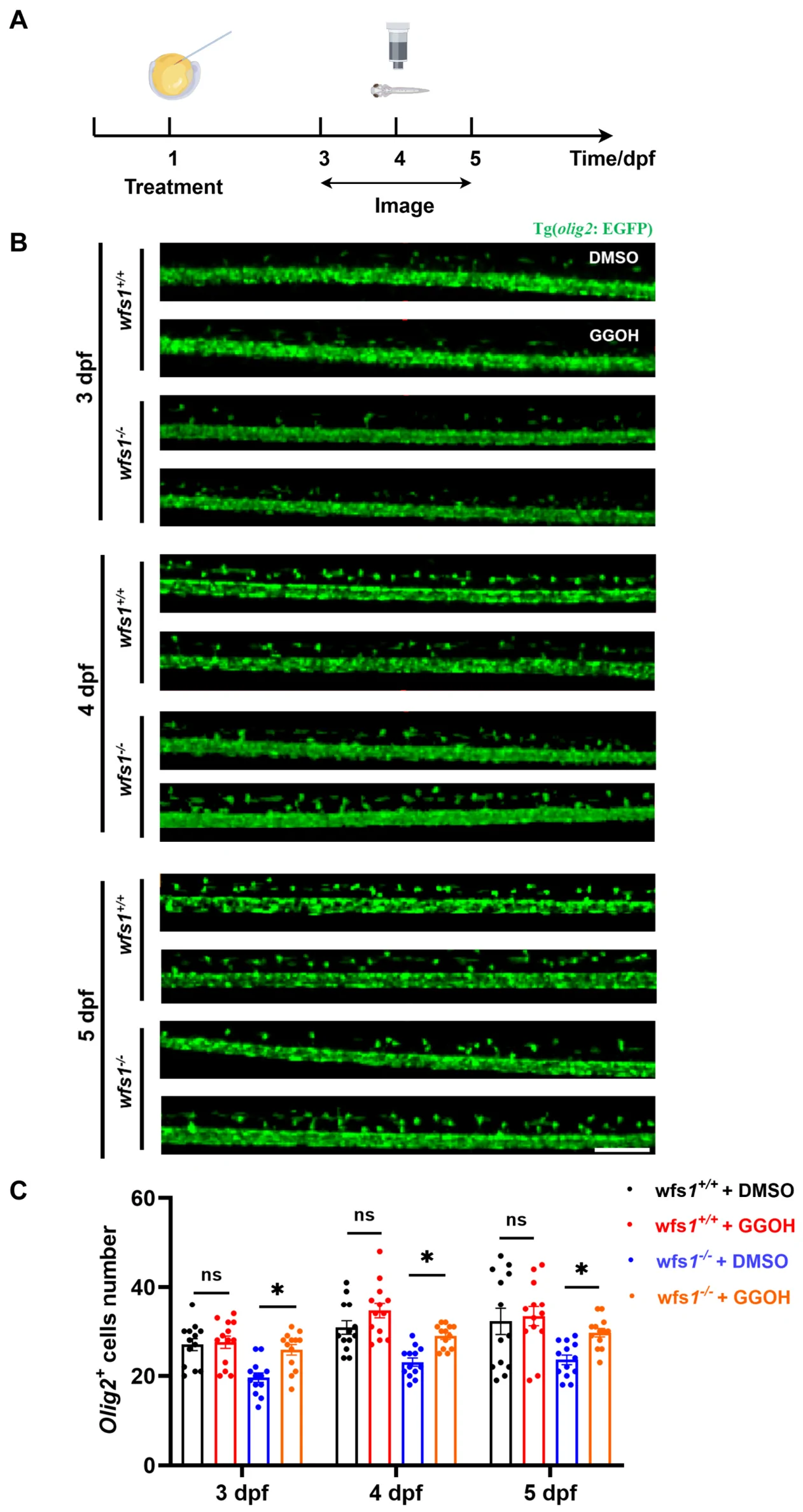
**GGOH specifically rescued OPC dorsal distribution defects in *wfs1^−/−^* zebrafish.** (**A**) Schematic of the experimental design. Yolk sac microinjection of GGOH or DMSO at 1 dpf in vivo tracking of dorsal distribution at 3–5 dpf. (**B**) Representative confocal images of *olig2^+^* cells migrating dorsally along Mauthner axons directly above the cloaca in zebrafish larvae at 3, 4, and 5 dpf. Scale bar: 20 μm. (**C**) Quantification of dorsally migrated OPCs. GGOH significantly restored the dorsal distribution in *wfs1^−/−^* group at 3–5 dpf after GGOH injection. Each dot represents one larva (n = 13 per group). Data are presented as mean ± SEM. Statistical analysis was performed using two-way repeated-measures ANOVA with group (genotype × treatment) as the between-subjects factor and timepoint (3, 4, 5 dpf) as the within-subjects factor, followed by Tukey’s multiple comparisons test. Within *wfs1^+/+^* larvae, DMSO vs. GGOH was not significant at any timepoint (*p* > 0.05). Within *wfs1^−^^/^^−^* larvae, DMSO vs. GGOH was significant at 3 dpf (*p* = 0.0244), 4 dpf (*p* = 0.0396), and 5 dpf (*p* = 0.0297). * *p* < 0.05; ns, not significant.

Previous studies have shown that microinjection of geranylgeraniol (GGOH) can reverse the migratory inhibition of OPCs caused by *hmgcs1* mutation by promoting protein geranylgeranylation [30,52,53]. Therefore, we conducted a pharmacological rescue experiment (Figure 6A). The intervention group received 1 nL of 1 M GGOH (dissolved in DMSO), while the control group received 1 nL of DMSO (vehicle control). After injection, the embryos were cultured until 3–5 dpf, and dorsal distribution of OPCs along the Mauthner axon was tracked in vivo using the Tg(*olig2*: EGFP) line live imaging (Figure 6B). The imaging results indicated that GGOH specifically reversed the OPC dorsal distribution delay caused by *wfs1* deficiency, with no significant effect observed in the *wfs1^+/+^* group, indicating that its action depends on the dysregulated *wfs1-hmgcs1* pathway (Figure 6C). These findings support the involvement of *hmgcs1*-dependent isoprenylation in the OPC phenotype of *wfs1* mutants. It should be noted that GGOH rescue was specific to OPC dorsal distribution; rescue of complete myelin structure was not assessed in this experiment and requires further investigation.

## 4. Discussion

As there is currently no cure for WS, therapeutic strategies accordingly focus on symptomatic management, aiming to delay complications and enhance the quality of life [4,11]. Gaining deeper insights into the phenotypes of WS complications and elucidating their underlying downstream mechanisms are of great importance. Consistent with previous neuroimaging findings of abnormal axonal myelination in the brain development of WS patients [2], this study revealed that *wfs1* deficiency reduces the dorsal distribution of OPCs along the zebrafish M-cell axon and supports a novel *wfs1*-*hmgcs1* association that may regulate dorsal distribution and myelination through isoprenylation.

Compared to mammalian models [28,54], zebrafish offer advantages of optical transparency, enabling real-time in vivo monitoring of OPC distribution during early development. Previous studies have shown that zebrafish with *wfs1* mutations are excellent animal models for studying WS, and share similar neurological pathologies that are observed in human WS patients, including progressive retinal ganglion cell loss and visual dysfunction [27,55]. Using CRISPR-Cas9 technology, we generated *wfs1* mutants in zebrafish, which exhibited systemic phenotypes consistent with WS, including reduced eye size, decreased heart rate, and significant motor dysfunction. These findings align with prior studies in murine models and clinical observations, confirming the utility of zebrafish for modeling WS-associated neurodevelopmental deficits. It should be noted, however, that the observed myelination defects arise in a whole-body knockout context, and the systemic phenotype, including reduced eye size, lower heart rate, and locomotor abnormalities, indicates general developmental perturbation. Therefore, we cannot exclude the possibility that the myelination abnormalities are secondary to developmental delay, general metabolic alterations, or other systemic effects. Future experiments using OPC-specific conditional knockout strategies will be necessary to determine whether *wfs1* acts cell-autonomously in oligodendrocytes.

Many studies have shown that WS can lead to sustained and severe ER stress. As the primary myelinating cells in CNS, oligodendrocytes are subjected to significant ER stress during the synthesis of large quantities of myelin membrane proteins, rendering them particularly vulnerable to ER stress [56]. Studies have revealed that ER stress is directly involved in the pathological processes of various myelin-related disorders, such as Pelizaeus–Merzbacher disease (PMD) [57] and Charcot–Marie–Tooth disease type 1A (CMT1A) [58], which is consistent with our results. Our findings revealed that *wfs1* deficiency disrupts OPC dorsal distribution and myelination, with transcriptomic evidence pointing to suppression of the *hmgcs1*-dependent isoprenylation pathway as a contributing mechanism. qRT-PCR analysis confirmed significant upregulation of classical ER stress markers such as *atf6*, *chop*, *hspa5*, and *xbp1s* in *wfs1^−/−^* larvae at 5 dpf, indicating that ER stress is concurrently activated. Whether the isoprenylation pathway acts independently of, downstream of, or synergistically with ER stress to impair OPC development remains to be determined; future studies employing ER stress inhibitors or OPC-specific transcriptomic profiling will be necessary to dissect the relative contributions of these pathways.

HMGCS1 is an upstream condensation enzyme in the mevalonate pathway, supplying substrate for HMGCR, the rate-limiting enzyme. Downstream products include cholesterol and isoprenoid intermediates that serve as essential lipid donors for the prenylation of small GTPases such as Rho and Ras [59]. HMGCS1-driven cholesterol production serves as a fundamental structural component for myelin membrane assembly and stability [60]. Mutation of the *hmgcs1* gene in zebrafish disrupts oligodendrocyte development and leads to aberrant myelination gene expression [30]. Similarly, we observed associated downregulation of *hmgcs1* in *wfs1* mutants, accompanied by reduced dorsal distribution of OPCs along the Mauthner axon and abnormal myelination development. A direct comparison with the *hmgcs1* mutant phenotype reported is informative [30]: in *hmgcs1* mutants, dorsal distribution arrest and myelin gene suppression were quantitatively similar in magnitude to those we observed in *wfs1^−^^/^^−^* fish, and GGOH rescue was effective in both models, further supporting the notion that *wfs1* deficiency acts at least partly through this pathway. However, whether *wfs1* directly regulates *hmgcs1* transcription or acts through intermediate ER stress-related or metabolic signals remains an important open question.

The partial recovery of myelin thickness at 8 dpf is an important finding that warrants careful interpretation. This recovery could reflect compensatory mechanisms at the level of individual axons in the sampled region, such as upregulation of alternative isoprenoid synthesis enzymes or delayed OPC maturation. Alternatively, it may reflect the spatially restricted nature of our Mauthner-axon-focused sampling, which may not represent global myelin status. The discrepancy between local recovery in zebrafish and the persistent developmental hypomyelination reported across multiple white matter tracts in WS patients [2,12] may also reflect genuine species differences in compensatory capacity. These considerations highlight the importance of the early developmental window as a therapeutic target, while also tempering direct translational extrapolation from zebrafish larvae to human disease.

However, several limitations of this study should be acknowledged. The *wfs1^−^^/^^−^* model is a whole-body knockout, and the observed systemic phenotypes indicate that myelination defects may be influenced by non-cell-autonomous mechanisms. Additionally, it is worth noting that the transgenic line Tg(*olig2*: EGFP) is a well-established and widely used tool in the zebrafish field, which has been extensively used in the field for FACS-based isolation of OPCs. Based on this precedent, we are confident that our sorting strategy effectively enriched the OPC population. However, FACS-sorted *olig2^+^* cells provided cell-type-enriched gene expression data, the Tg(*olig2*: EGFP) line labels the broader oligodendrocyte lineage including both OPCs and mature oligodendrocytes, and sorted populations were not further validated by independent markers such as *sox10* or secondary immunostaining; therefore, contamination from other *olig2*-expressing cell types cannot be entirely excluded. The precise molecular interaction between *wfs1* and *hmgcs1* has not been elucidated; it remains unclear whether *wfs1* directly regulates *hmgcs1* transcription or acts through intermediate signals. The contributions of other glial cell types, such as astrocytes and microglia, which are also known to influence myelination and myelin repair, have not been explored in this context. It would be interesting to understand how *wfs1* deficiency affects the broader glial environment. In addition, we have not yet been able to delineate which specific downstream GTPases are affected by impaired isoprenylation. Furthermore, although ER stress activation was confirmed at the whole-larva level, the relative contribution of ER stress versus isoprenylation deficiency to the OPC-specific phenotypes has not been dissected; cell-type-specific analyses and pathway inhibitor experiments will be needed to address this question. In the future, it would be very valuable to determine whether OPC-autonomous loss of *wfs1* is sufficient to recapitulate the myelination defects observed here, using conditional knockout strategies, and to employ single-cell RNA sequencing to dissect OPC heterogeneity under *wfs1* deficiency.

The present study also has implications for therapeutic strategy. Given that GGOH supplementation specifically restored OPC dorsal distribution in *wfs1^−^^/^^−^* zebrafish, targeting the isoprenylation pathway may represent a tractable approach for early intervention in WS-associated hypomyelination. However, since the current evidence is derived from zebrafish larvae and does not yet encompass mammalian models or complete myelin rescue, translation to clinical application requires significant further investigation.

## 5. Conclusions

This study suggests that *wfs1* deficiency delays OPC dorsal distribution and early myelination in zebrafish, partly associated with suppression of *hmgcs1*-dependent isoprenoid metabolism. GGOH rescue supports involvement of the isoprenylation pathway in dorsal distribution defects, although direct regulation of *hmgcs1* by *wfs1* and cell-autonomous mechanisms in oligodendrocytes require further investigation. By integrating in vivo imaging, transcriptomics, and pharmacological rescue, we provided evidence that early myelination defects in *wfs1*-deficient zebrafish are associated with changes in OPC-associated glial metabolism, potentially including both cell-autonomous and systemic contributions. These findings expand the pathological spectrum of WS and nominate *hmgcs1* and protein prenylation as candidate therapeutic targets for WS-related hypomyelination, pending validation in mammalian models.

## Data Availability

All data generated or analyzed during this study are included in this published article.

## Supplementary Materials

The following supporting information can be downloaded at: https://www.mdpi.com/article/10.3390/biology15161402/s1, Supplementary Table S1: Plasmid constructs primer sequences; Supplementary Figure S1: Quantification of eye area in *wfs1^−/−^* zebrafish larvae; Supplementary Figure S2: OPCs rarely undergo apoptosis in *wfs1^−/−^* larvae at 3 and 5 dpf; Supplementary Figure S3: The hypomyelination phenotype in *wfs1^−/−^* mutants emerges during early developmental stages and recovers by 8 dpf; Supplementary Figure S4: ER stress markers are upregulated in *wfs1^−/−^* zebrafish at 5 dpf; File S1. Transcriptome sequencing data and differentially expressed genes (DEGs) in *wfs1^−/−^* zebrafish at 5 dpf; File S2: The original Western blot images.

## Abbreviations

The following abbreviations are used in this manuscript:

Abbreviation	Full Name
ANOVA	Analysis of variance
CNS	Central nervous system
CRISPR-Cas9	Clustered Regularly Interspaced Short Palindromic Repeats-Cas9
Ct	Cycle threshold
cDNA	Complementary DNA
DEGs	Differentially expressed genes
DMSO	Dimethyl sulfoxide
dpf	Days post-fertilization
ECL	Enhanced chemiluminescence
EGFP	Enhanced Green Fluorescent Protein
ER	Endoplasmic reticulum
FACS	Fluorescence-Activated Cell Sorting
FSC	Forward scatter
GGOH	Geranylgeraniol
GO	Gene Ontology
*hmgcs1*	3-hydroxy-3-methylglutaryl-CoA synthase 1
hpf	Hours post-fertilization
HRP	Horseradish peroxidase
KEGG	Kyoto Encyclopedia of Genes and Genomes
M-cells	Mauthner cells
MS222	Tricaine methanesulfonate
OPCs	Oligodendrocyte precursor cells
PBS	Phosphate-buffered saline
PMD	Pelizaeus–Merzbacher disease
PTU	N-phenylthiourea
PVDF	Polyvinylidene fluoride
qRT-PCR	Quantitative real-time PCR
RNA-seq	RNA sequencing
SDS-PAGE	Sodium Dodecyl Sulfate-Polyacrylamide Gel Electrophoresis
SEM	Standard error of the mean
sgRNA	Single-guide RNA
SSC	Side scatter
SYBR Green	SYBR Green fluorescent dye
TBST	Tris-Buffered Saline with Tween
TEM	Transmission electron microscopy
*wfs1*	Wolfram syndrome 1
WS	Wolfram syndrome
WT	Wild-type
